# Surgical management for isolated macrodactyly in an adult PIK3CA mutant

**DOI:** 10.1016/j.jpra.2020.10.002

**Published:** 2020-10-22

**Authors:** C.J. Jacobs, M. Vreeburg, C.E.M. de Die-Smulders, H.M. Staal, T. Lauwers

**Affiliations:** a Faculty of Health, Maastricht University, Medicine and Life Sciences, the Netherlands; b Department of Plastic, Reconstructive and Hand Surgery, Maastricht University Medical Centre+, MUMC+, Box 5800, 6202 AZ Maastricht, the Netherlands; c Department of Clinical Genetics, MUMC+, Maastricht, the Netherlands; d School for Oncology & Developmental Biology (GROW), Maastricht University, the Netherlands; e Department of Orthopaedics, MUMC+, Maastricht, the Netherlands

**Keywords:** Macrodystrophia lipomatosa, Macrodactyly, PIK3CA mutation, Partial amputation, Carpal bones stability

## Abstract

Isolated macrodactyly in adults caused by mosaic pattern PIK3CA mutation can result in significant functional impairment and psychological burden. Due to the rarity of this condition there are no clear treatment guidelines, and those few available are focused on paediatric cases. Reports on surgical management of isolated macrodactyly in adults are lacking. We present here the surgical management through partial amputation of enlarged rays of the right hand in an individual affected by low-grade mosaic PIK3CA mutation.

## Introduction

We present a case of surgical management for enlargement of the thumb and index rami (1st & 2nd) of the right hand in a 55-year-old female patient with a low-grade mosaic pattern PIK3CA mutation. The challenge in advanced cases such as this is to maintain the stability of the wrist and hand while preserving a maximum of mobility and function.

## Case description

A 53-year-old female patient presented to our centre for consultation due to progressive isolated gigantism of the right hand paired with pain and loss of function. She had no family history of genetic or congenital malformations. Enlargement of right-hand thumb, index and middle (1st, 2nd & 3rd) digits had been noticed at 6 months of age, with progressive growth leading to functional disability.

At first presentation to our centre, she had previously undergone eight surgical debulking procedures of the enlarged digits throughout her lifetime. Medical treatment with growth-inhibitors was also attempted yet ineffective. The patient had initially been diagnosed with Proteus syndrome based on clinical presentation. Due to uncertainty of this diagnosis and patient's request, genetic testing was conducted through biopsies from enlarged tissues which confirmed a low-grade mosaic mutation in the PIK3CAgene.

Symptoms experienced at this stage were pain and loss of function in the right hand with lack of proper grip function. Physical examination showed tissue enlargement, predominantly around the right wrist and volar aspect of the thumb. The wrist was furthermore hyperextended with the thumb and index digits in a fixated position.

Having been informed of risks of surgery, the patient initially selected an expectative approach. However, due to increasing impairment and vascular compromise, the patient ultimately chose to undergo surgical intervention.

### Surgical intervention

As debulking was not possible at this stage, we performed a partial amputation of the enlarged tissues along with wrist stabilization through the superficial and deep digital flexor tendons to the index digit (hereafter referred to as FDS2 and FDP2, respectively). *Supplementary Digital Materials (SDM) 1 & 2* showcase preoperative external and radiographic view of the affected area. The procedure went as follows:

Incisions were planned to conserve as much skin as possible, mainly on the dorsal aspect.

The median nerve was identified and showed extreme enlargement and ulnar deviation caused by a radial exostosis. Numerous (>15) large and rigid branches to the thenar were encountered and severed during further dissection. Branches to the middle and ring rami were spared and showed to normalize along their distal course. However, at wrist level the entire median nerve showed to be hypertrophic.

A skin flap on the ulnar side of the proximal index digit was spared including the neurovascular bundle containing a hypertrophic collateral nerve, which was removed to minimize recurrence risk.

Thenar muscles also showed hypertrophy, whereas flexor pollicis longus (FPL) and both the FDS2 and FDP2 flexor tendons appeared normally sized. The tendons were further dissected and severed at the level of their insertion. The median nerve normalized in size during its’ proximal course at the proximal wrist crease.

**Figure 1:**
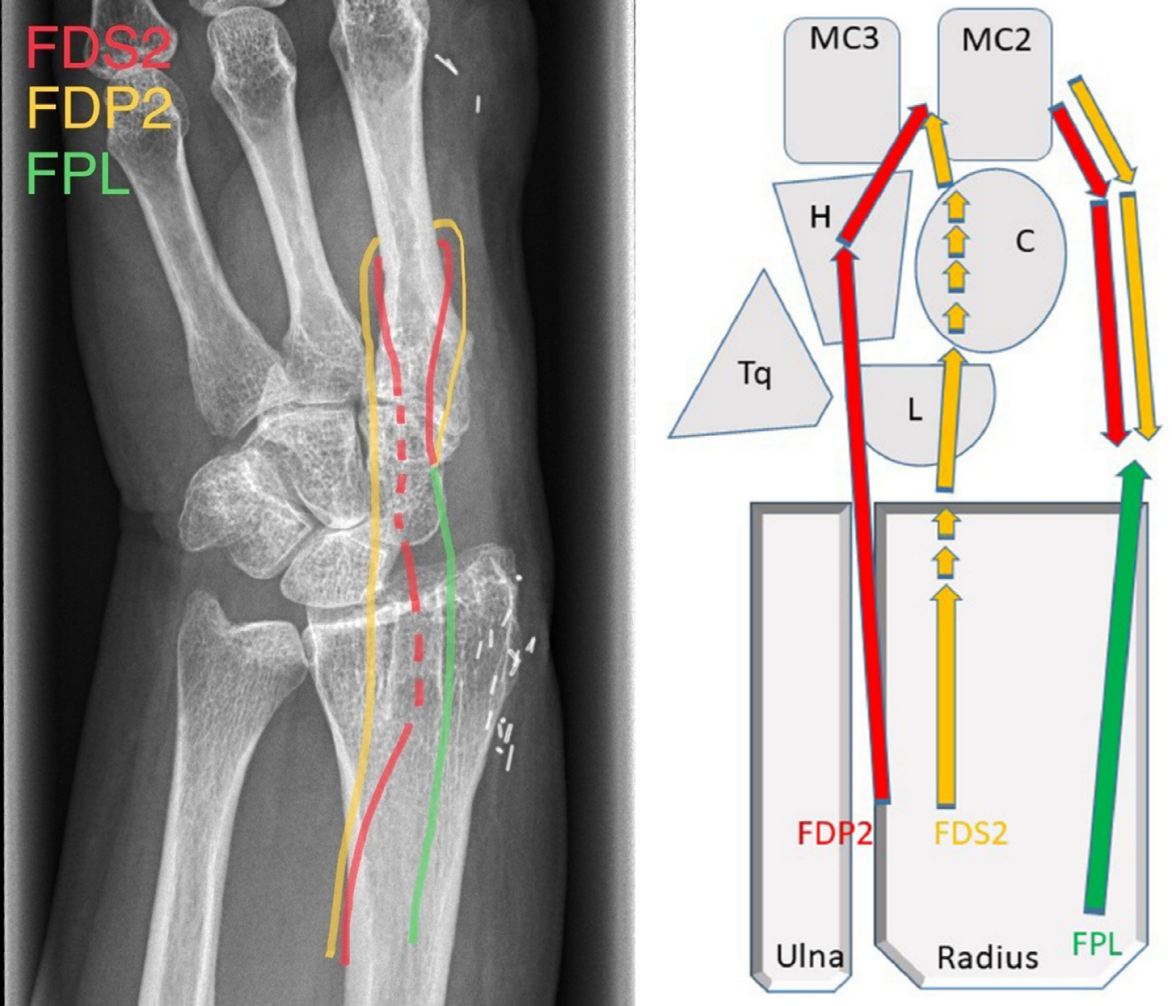
Schematic figure illustrating stabilisation technique through transosseous ligament reconstruction of FDP2 (Flexor Digitorum Profundis of 2nd digit), FDS2 (Flexor Digitorum Superficialis of 2nd digit) and FPL (Flexor Pollicis Longus).

The amputation followed in which the thumb and index rami, Trapezium, Trapezoid and Scaphoid bones (STT) were all removed. An exostosis on the palmar side of the Capitate bone was also removed, as well as a large exostosis on the palmar Radius. The Lunate bone luxated easily out of the Lunate facet on the Radius while stability of the third metacarpal-capitate-lunate joints appeared to be preserved. A canal was drilled through Radius, Capitate and base of the middle (3rd) metacarpal (MC3) through which the FDS2 was passed, after which the FDP2 was guided around the MC3 to serve as a static wrist stabilizer. The tendons were sutured onto the FPL. This was a tailored solution for the radiocarpal instability that occurred after resection of the STT bones, combined with the resection of the palmar part of radius including the palmar capitate. This equals resection of the complex with the radio-carpal ligaments attached and loss of the radiolunate, radioscapholunate, radioscaphocapitate and radial collateral ligaments. This resulted in gross instability of the wrist with a spontaneous radiodorsal luxation of the lunate and remnant of the radiocarpal joint. The transosseus ligament reconstruction secured a functionally mobile and stable wrist as can be seen in the video. The spared dorsal skin flap was used for wound closure through staples and ethilon sutures. The technique for wrist stabilisation has been depicted schematically Fig. 1.

**Figure 2:**
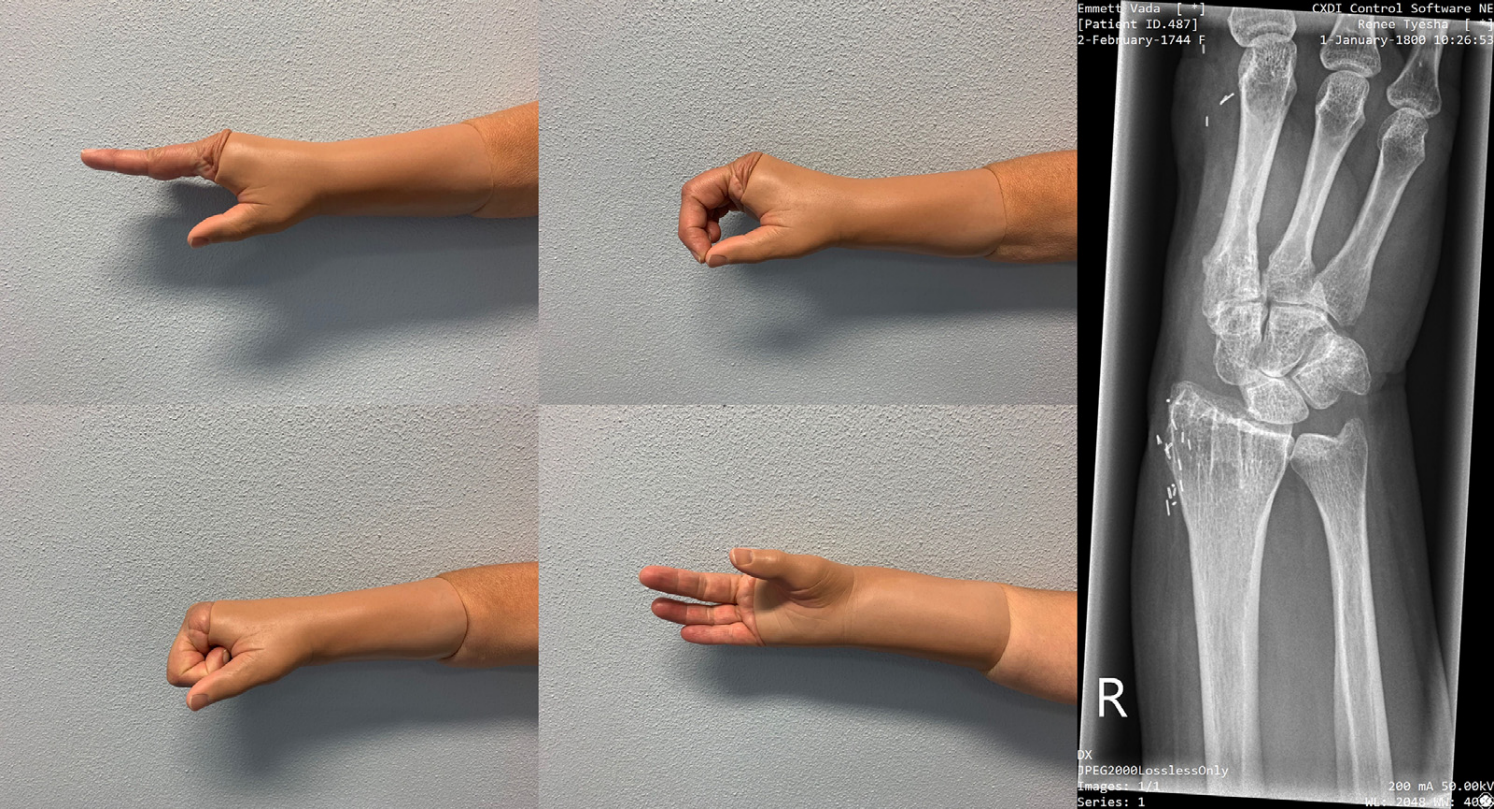
Illustration on postoperative radiograph depicting stabilisation technique through transosseous ligament reconstruction of FDP2 (Flexor Digitorum Profundis of 2nd digit), FDS2 (Flexor Digitorum Superficialis of 2nd digit) and FPL (Flexor Pollicis Longus).

Postoperative treatment consisted of temporary splinting and physiotherapy. Besides phantom pain postoperative course was unremarkable during the two year follow up. Videos 1 and 2 (available as supplemental digital content) showcase the postoperative function at this stage. The patient preserved excellent function of her right hand. Photographs taken at three years post-operatively depict range of motion and aesthetic result (*SDM 3–7)*. Follow up X-ray photographs taken two and three years post-operatively (*SDM 8 & 9)* showed unchanged position of the MC3-Capitate-Radial alignment compared to immediate post-operative X-ray (*SDM 10)*, and clinical stability was unchanged at two- and three-year postoperative examinations Fig. 2.

## Discussion

### Genetic basis of macrodactyly

Isolated macrodactyly has been reported as a rare congenital anomaly linked to several mosaic mutations.^1^ These overgrowth syndromes have been shown to be caused by somatic mutations in the P13K/PTEN/AKT/TSC/mTORC1 signalling pathways.^2^ These somatic mosaic mutations have been found in biopsies retrieved from affected tissues.^1^^,^^3^, ^4^, ^5^, ^6^, ^7^

Based on the causative genetic mutation, overgrowth syndromes can be divided into: Proteus Syndrome (PS) which is caused by mosaic mutations in the AKT1 gene, PIK3CA-related overgrowth spectrum (PROS) which is caused by mutations in the PIK3CA oncogene, and PTEN hamartoma tumour syndrome (PHTS): caused by somatic PTEN tumour suppressor gene mutations.^1^^,^^2^

As clinical manifestations often overlap, genetic testing is crucial in accurate diagnosis of individuals suffering from these overgrowth syndromes. Accurate genetic diagnosis is of clinical importance for non-surgical medical management with targeted growth inhibiting drugs and thus plays a role in therapeutic intervention prior to surgery.^1^

### Clinical presentation

The condition is represented by benign overgrowth of osseous, adipose, nervous and/or cutaneous tissue causing disproportionate enlargement and loss of function of the affected area. The hypertrophy is typically found within an area of nerve distribution with the nerve in question also being enlarged and/or elongated.^4^^,^^8^ Most frequently, the terminal branches of the median nerve are affected.

### Management

Due to the rarity of the condition, there are no clear guidelines for surgical management of progressive and isolated macrodactyly. There are a handful of publications which describe surgical management and therapeutic consideration for macrodactyly.^6^, ^7^, ^8^ These reports mostly describe paediatric cases, however. Cases of surgical management for fully developed and previously untreated macrodactyly in adulthood or older children have also been described.^9^ To our knowledge, reports of adults affected by macrodactyly with several previous surgical interventions are few in number.

Possible interventions described in these reports are surgical debulking of enlarged soft tissue, osteotomies, and complete amputation of affected area have been described for macrodactyly in infants and children.^6^, ^7^, ^8^ The importance of individualized approaches is emphasized, with amputation as an important intervention to consider in severe/progressive macrodactyly cases. In cases like this one with a resection of bone and attached ligaments at the level of the wrist, the recreation of a stable wrist joint is paramount.

### Recommendations

Our case describes successful management and gain of function after partial amputation of progressive macrodactyly in an adult patient who had previously undergone several surgical interventions. Although this can be psychologically challenging in an older patient, partial amputation of the wrist risks resulting in gross instability. Tailored solutions can bring a functionally acceptable result for adult PIK3CA patients with progressive and functionally impairing macrodactyly.

## Appendix Supplementary materials

Image, application 1

Image, video 2

Image, video 3
