# Supplementary material for: Surgical management for isolated macrodactyly in an adult PIK3CA mutant
Source: JPRAS Open. 2020 Oct 22;26:86–90. doi: 10.1016/j.jpra.2020.10.002 (PMC7680883; doi:10.1016/j.jpra.2020.10.002)

Supplementary Digital Material (SDM) legends:

SDM 1: Preoperative view of the affected hand.


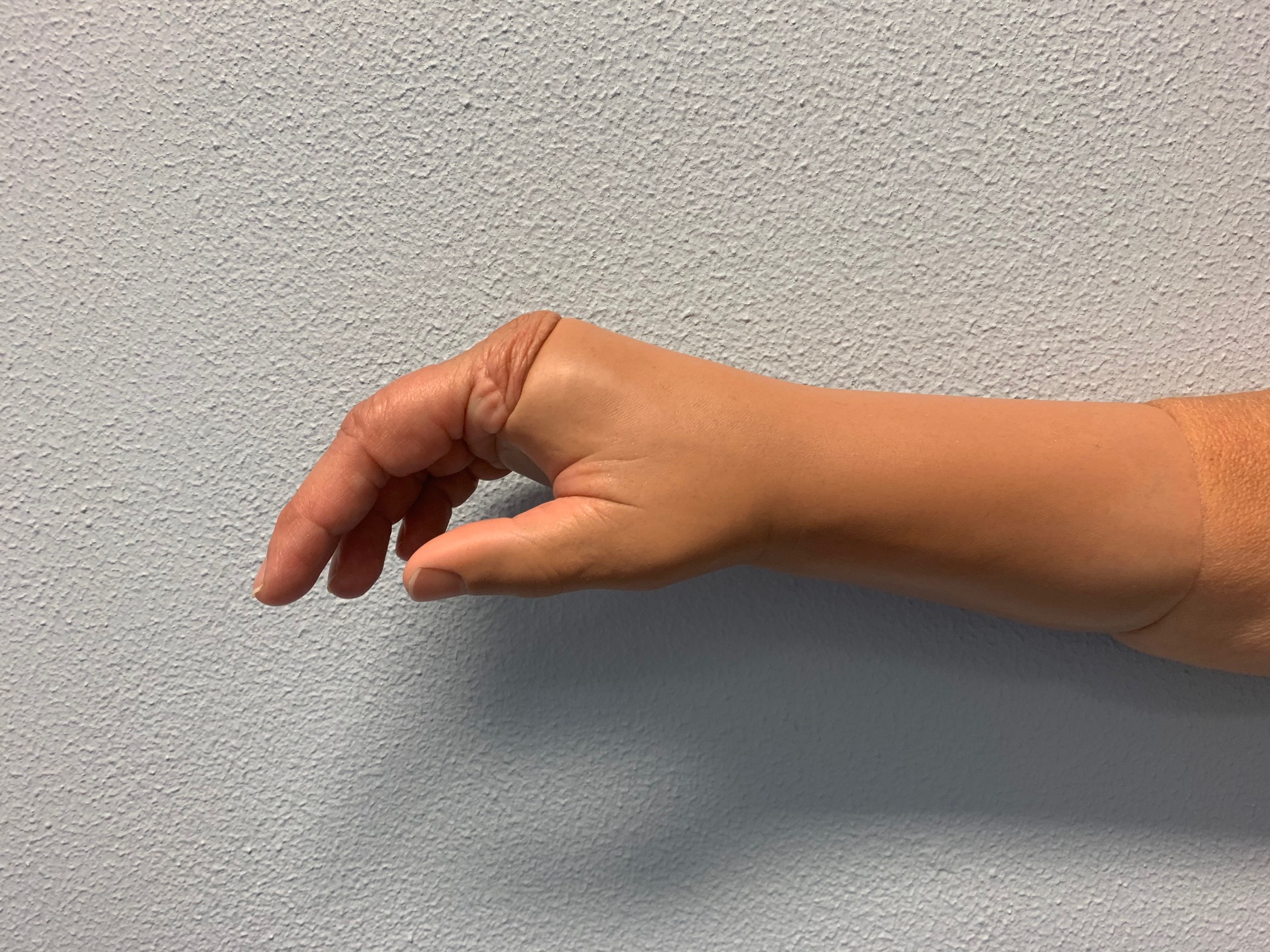


SDM 2: Preoperative radiograph of affected hand.


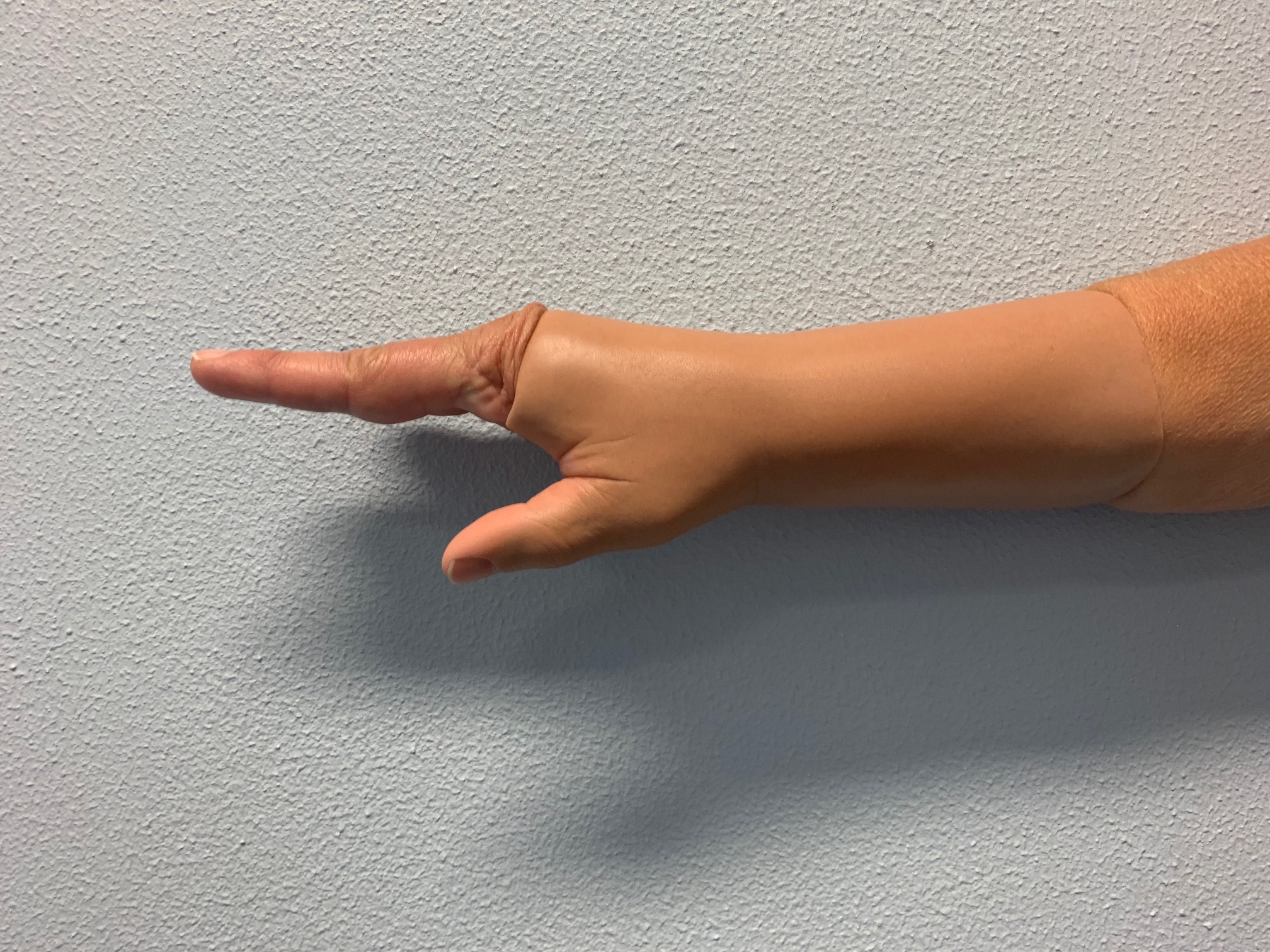


SDM 3-7: Depicted range of Motion and aesthetic result achieved with prosthesis three years post-operatively.


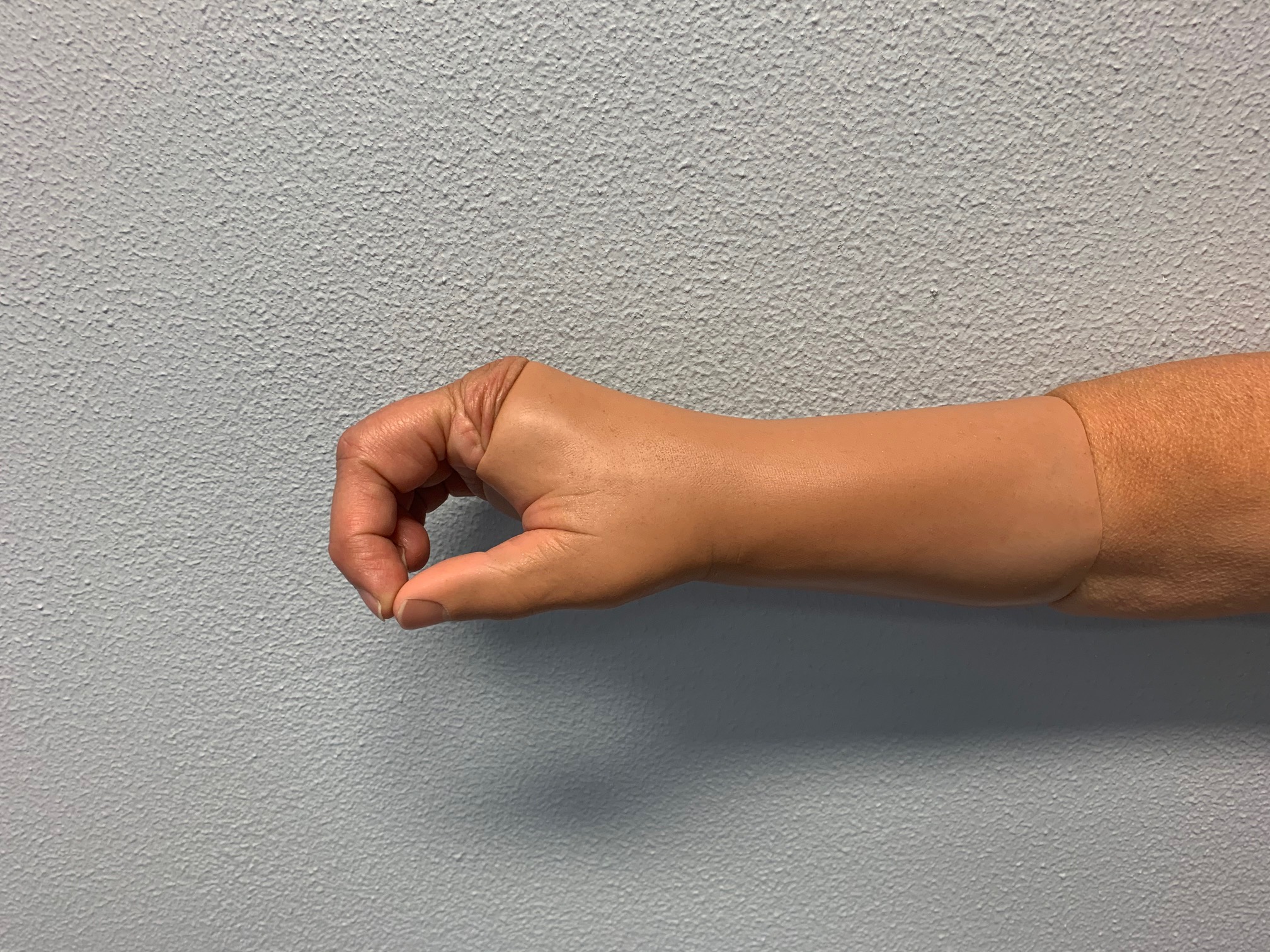

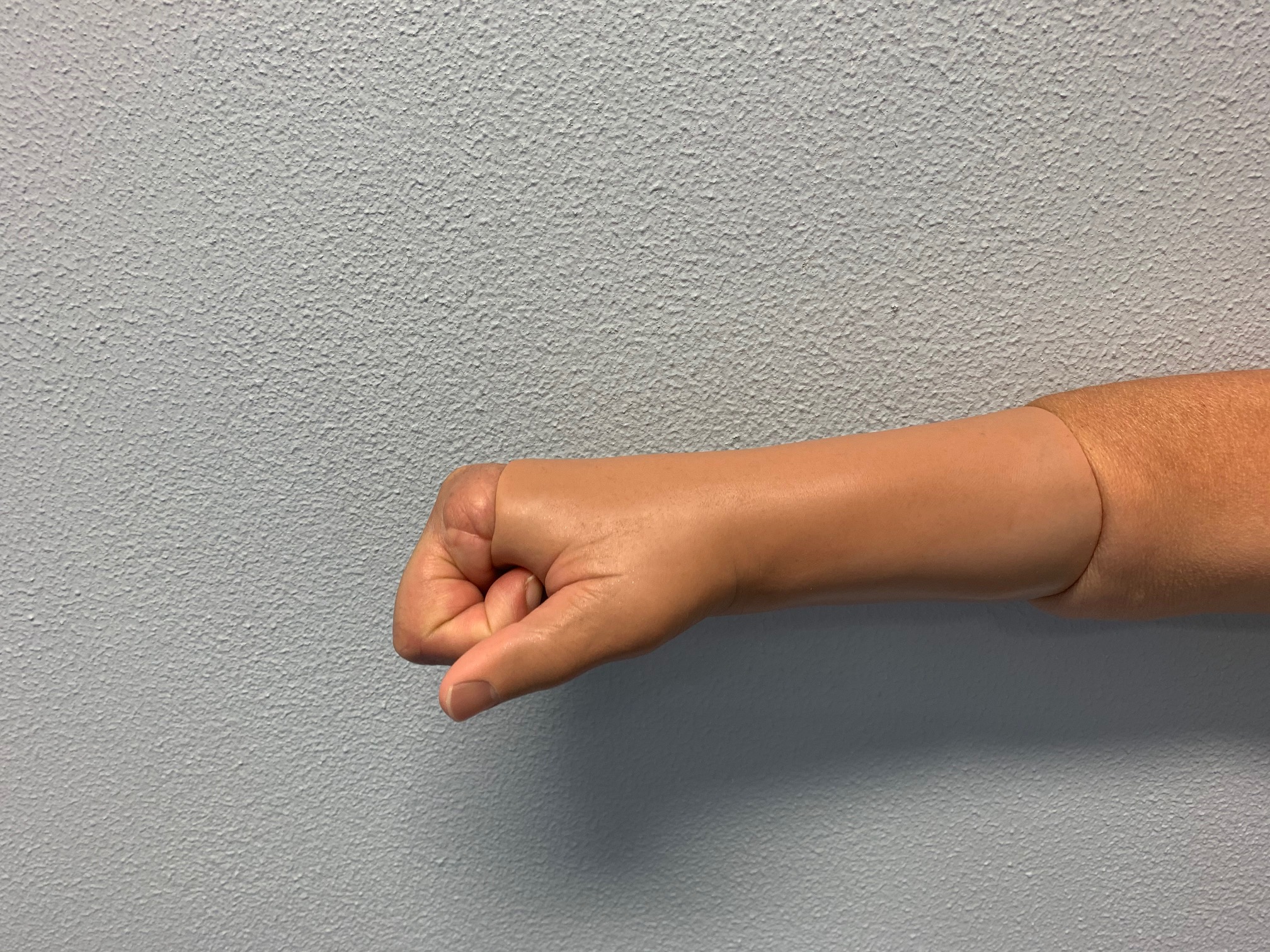

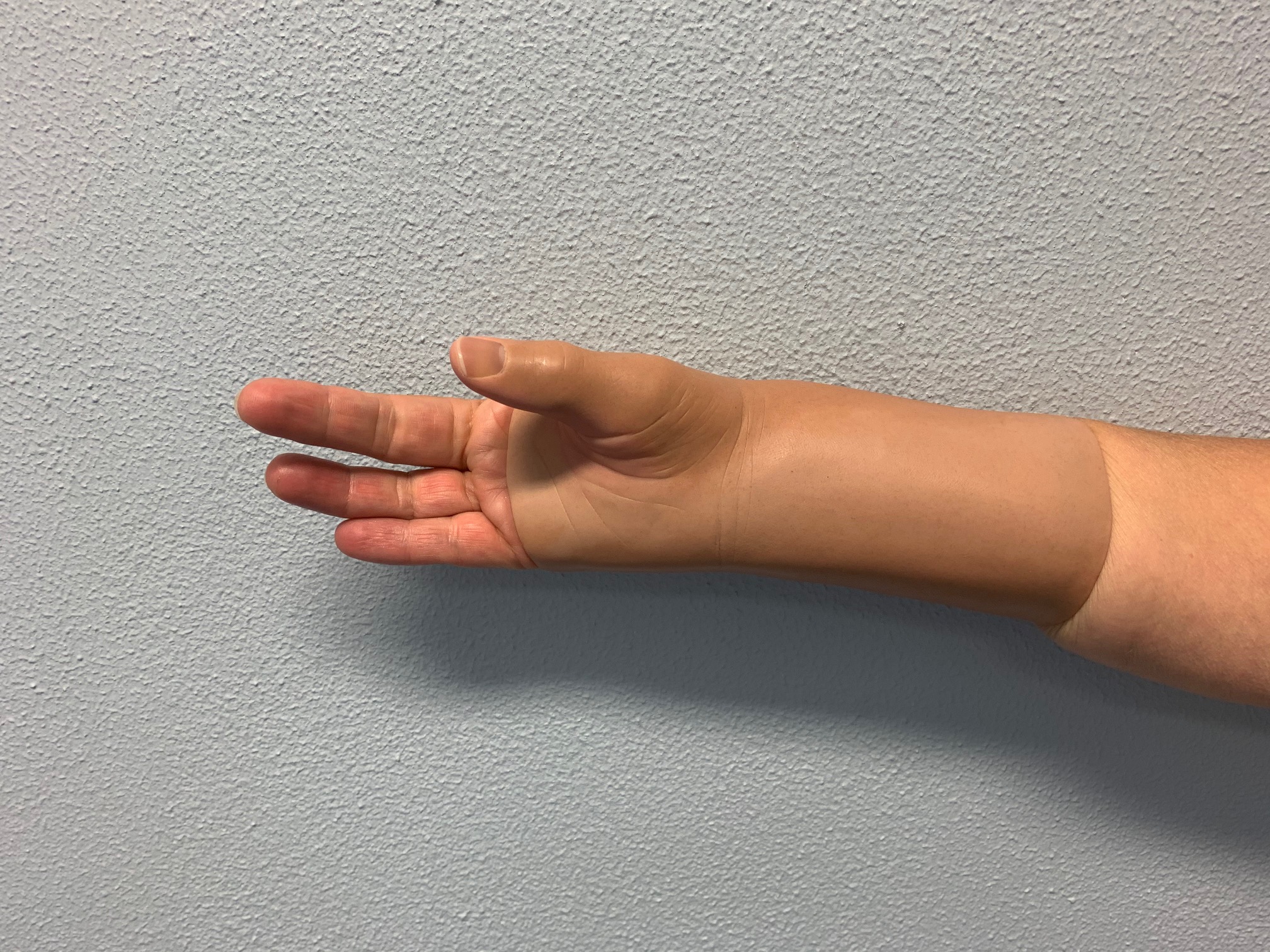

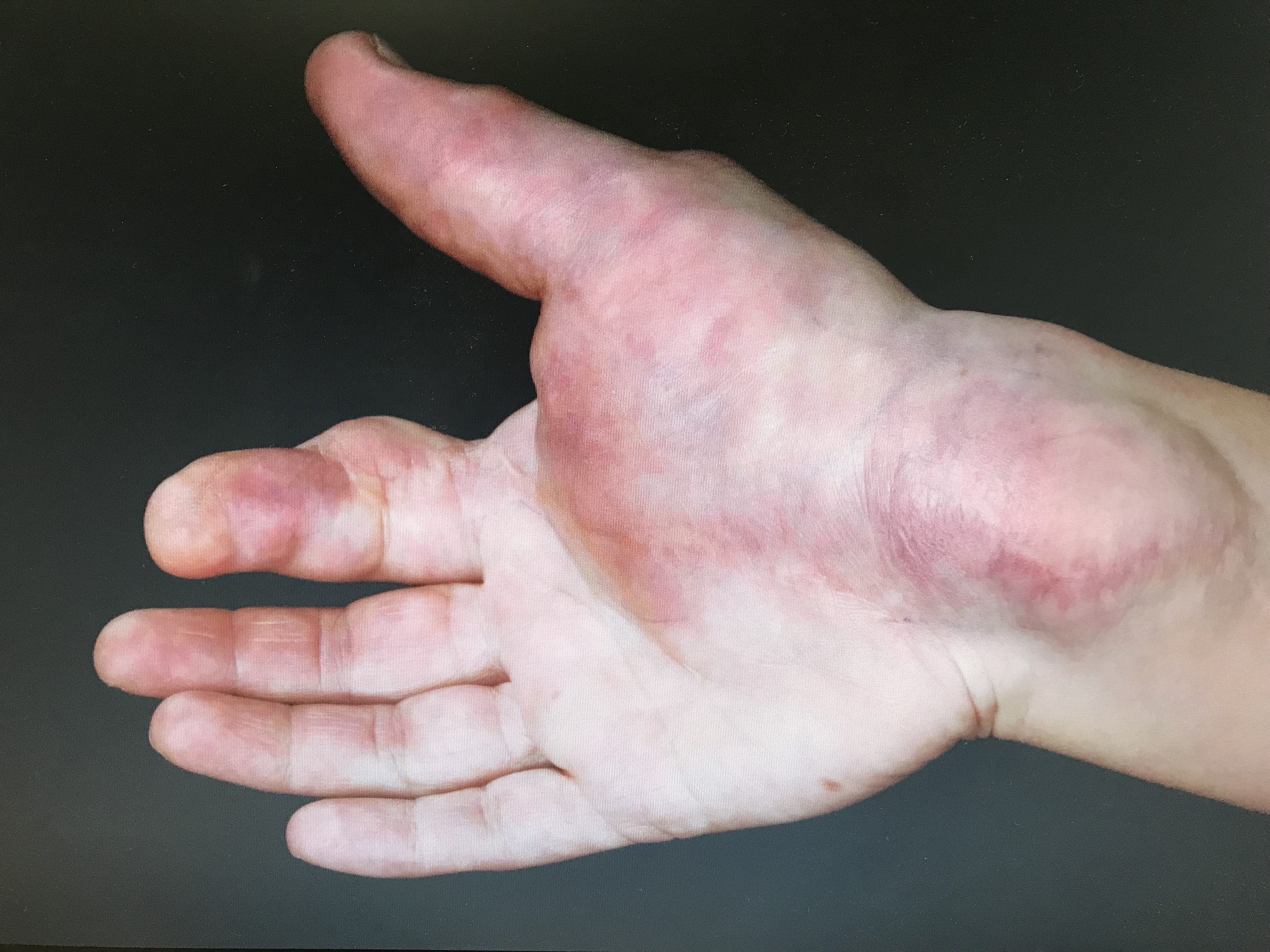

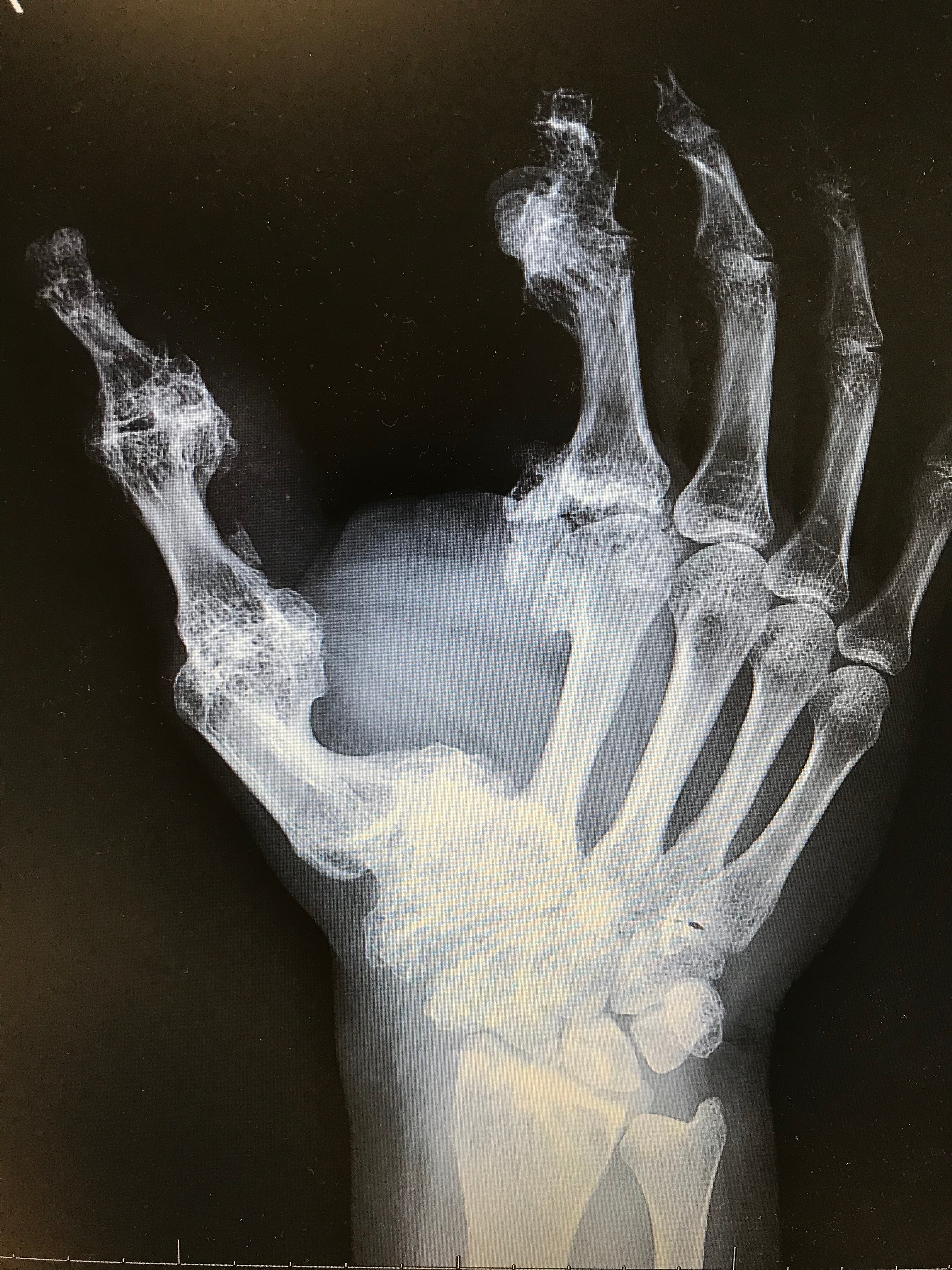


SDM 8: Radiograph taken two years post-operatively. Please note: the information and dates on this image are randomly computer-generated text added when anonymously downloading images from our hospital’s electronic imaging system. This information does not reflect any real patient data.


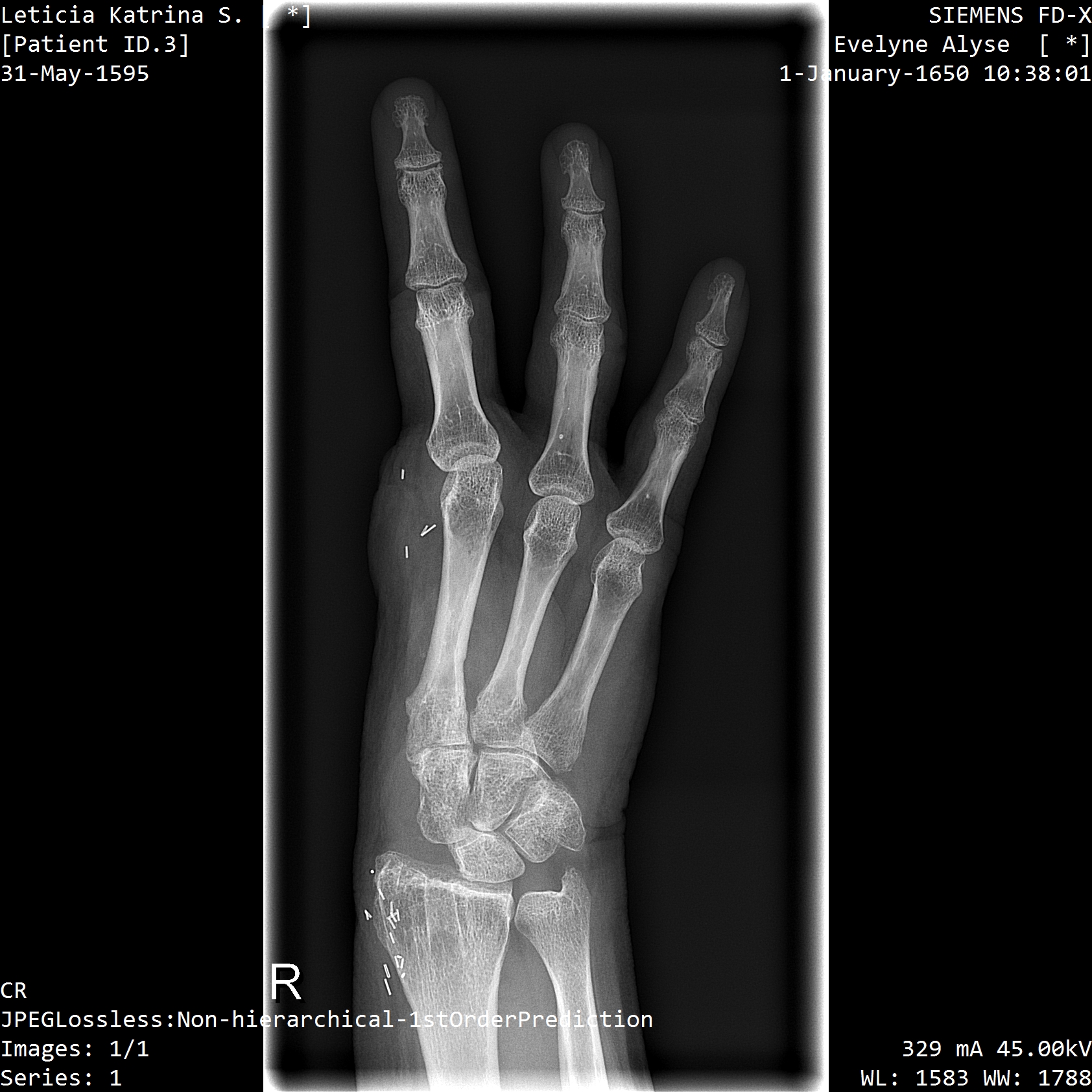


SDM 9: Radiograph taken three years post-operatively. Please note: the information and dates on this image are randomly computer-generated text added when anonymously downloading images from our hospital’s electronic imaging system. This information does not reflect any real patient data.


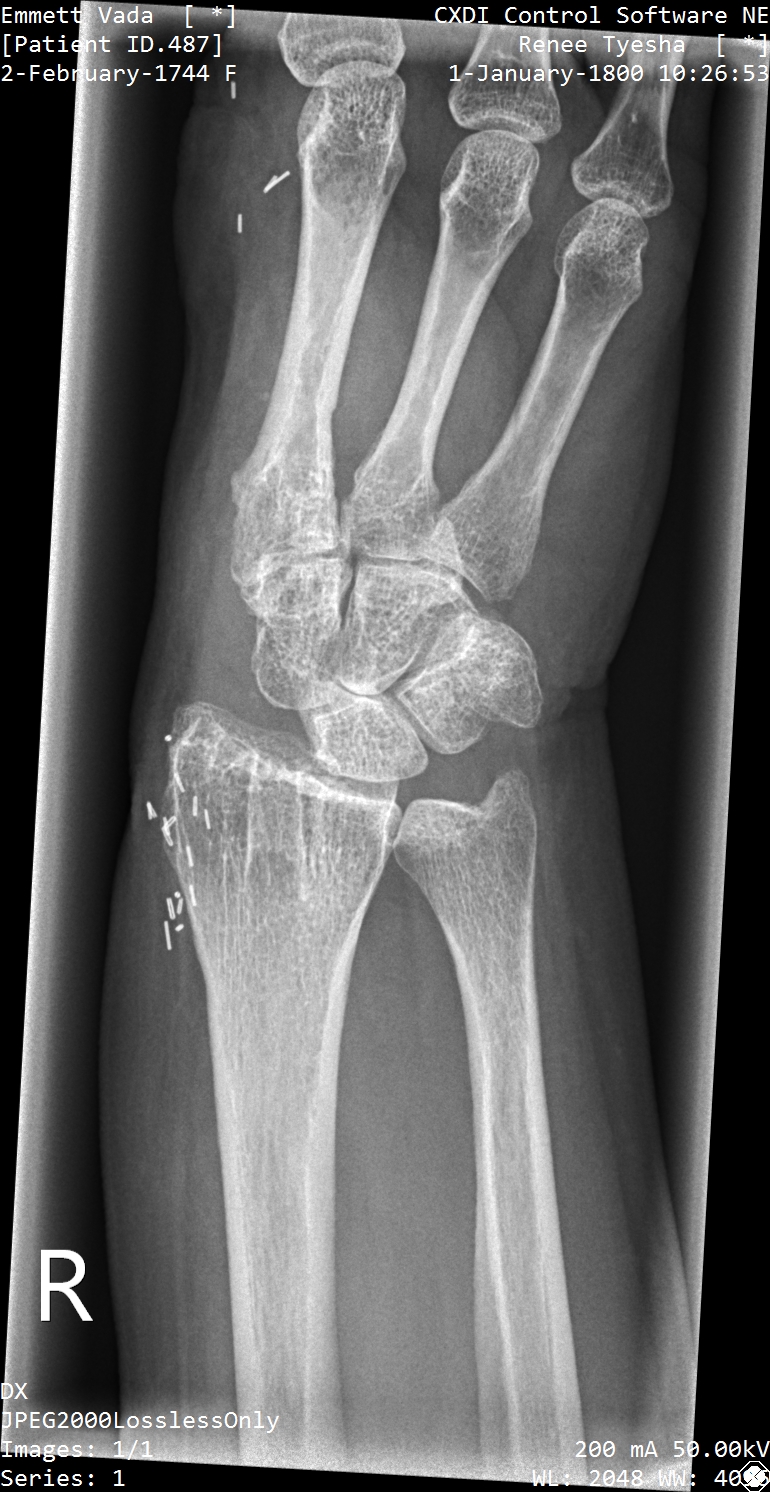


SDM 10: Radiograph taken immediately post-operatively.


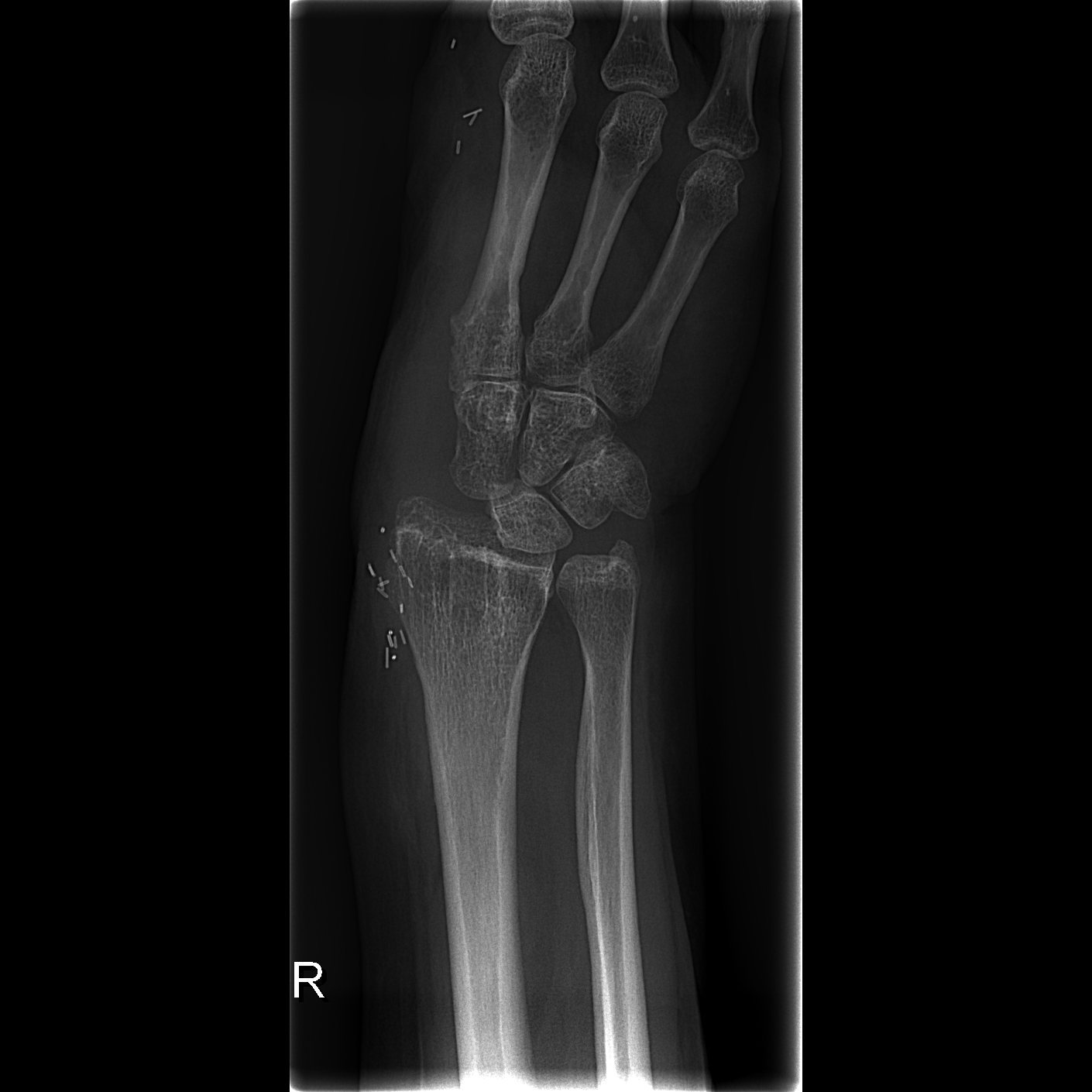

Supplement: Supplementary file 1 [file mmc1.docx]
